# Genetic testing and surveillance in infantile myofibromatosis: a report from the SIOPE Host Genome Working Group

**DOI:** 10.1007/s10689-020-00204-2

**Published:** 2020-09-05

**Authors:** Simone Hettmer, Guillaume Dachy, Guido Seitz, Abbas Agaimy, Catriona Duncan, Marjolijn Jongmans, Steffen Hirsch, Iris Kventsel, Uwe Kordes, Ronald R. de Krijger, Markus Metzler, Orli Michaeli, Karolina Nemes, Anna Poluha, Tim Ripperger, Alexandra Russo, Stephanie Smetsers, Monika Sparber-Sauer, Eveline Stutz, Franck Bourdeaut, Christian P. Kratz, Jean-Baptiste Demoulin

**Affiliations:** 1 Division of Pediatric Hematology and Oncology, Department of Pediatric and Adolescent Medicine, University Medical Center Freiburg, University of Freiburg, Freiburg, Germany; 2 De Duve Institute, University of Louvain, Brussels, Belgium; 3 Department of Pediatric Surgery, University Hospital Gießen-Marburg, Marburg, Germany; 4 Institute of Pathology, Friedrich‐Alexander‐University Erlangen‐Nürnberg, University Hospital, Erlangen, Germany; 5 Division of Pediatric Oncology, Great Ormond Street Hospital, London, Great Britain UK; 6 Princess Máxima Center for Pediatric Oncology, Utrecht, The Netherlands; 7 Department of Genetics, University Medical Center Utrecht/Wilhelmina Children′s Hospital, Utrecht, The Netherlands; 8 Institute of Human Genetics, Heidelberg University Hospital, Heidelberg, Germany; 9 Hopp Children’s Cancer Center Heidelberg (KiTZ), Heidelberg, Germany; 10 Division of Pediatric Oncology, Sheba Medical Center, Ramat Gan, Israel; 11 Department of Pediatric Hematology/Oncology, Universitätsklinikum Hamburg-Eppendorf, Hamburg, Germany; 12 Department of Pathology, University Medical Center Utrecht, 3584 CX Utrecht, The Netherlands; 13 Department of Pediatric and Adolescent Medicine, Children’s Hospital, Friedrich‐Alexander‐University Erlangen‐Nürnberg, Erlangen, Germany; 14 Division of Hematology/Oncology, Schneider Children’s Medical Center of Israel, Petah Tikva, Israel; 15 Swabian Children’s Cancer Center, Children’s Hospital Augsburg, Augsburg, Germany; 16 Department of Clinical Genetics, Uppsala University Hospital, Uppsala, Sweden; 17 Department of Immunology, Genetics and Pathology, Uppsala University, Uppsala, Sweden; 18 Department of Human Genetics, Hannover Medical School, Hannover, Germany; 19 Section of Pediatric Oncology, Children’s Hospital, University Medical Center of the Johannes Gutenberg University Mainz, Mainz, Germany; 20 Olgahospital, Klinikum Stuttgart, Stuttgart, Germany; 21 Pediatric Oncology, University Children’s Hospital Zürich, Zürich, Switzerland; 22 SIREDO Pediatric Cancer Center, Institute Curie, Paris, France; 23 Division of Pediatric Hematology and Oncology, Hannover Medical School, Hannover, Germany

**Keywords:** Infantile myofibromatosis, PDGFRB variants, Genetic counseling, Surveillance

## Abstract

Infantile myofibromatosis (IM), which is typically diagnosed in young children, comprises a wide clinical spectrum ranging from inconspicuous solitary soft tissue nodules to multiple disseminated tumors resulting in life-threatening complications. Familial IM follows an autosomal dominant mode of inheritance and is linked to *PDGFRB* germline variants. Somatic *PDGFRB* variants were also detected in solitary and multifocal IM lesions. *PDGFRB* variants associated with IM constitutively activate PDGFRB kinase activity in the absence of its ligand. Germline variants have lower activating capabilities than somatic variants and, thus, require a second cis-acting hit for full receptor activation. Typically, these mutant receptors remain sensitive to tyrosine kinase inhibitors such as imatinib. The SIOPE Host Genome Working Group, consisting of pediatric oncologists, clinical geneticists and scientists, met in January 2020 to discuss recommendations for genetic testing and surveillance for patients who are diagnosed with IM or have a family history of IM/*PDGFRB* germline variants. This report provides a brief review of the clinical manifestations and genetics of IM and summarizes our interdisciplinary recommendations.

## Introduction

Infantile myofibromatosis/myofibromas (IM) were first described by Stout in 1954 as congenital generalized fibromatosis [1]. Disease manifestations range from solitary soft tissue nodules (infantile myofibromas) to multiple or disseminated (generalized) tumors (infantile myofibromatosis) with life-threatening complications, particularly if visceral disease is present. Most IM cases are diagnosed in children below 2 years of age. The reported incidence of IM is 1 in 150,000 live births, but, as minor forms of the disease may go unnoticed, the true incidence of IM is likely much higher [2].

## Clinical case presentation

**Fig. 1:**
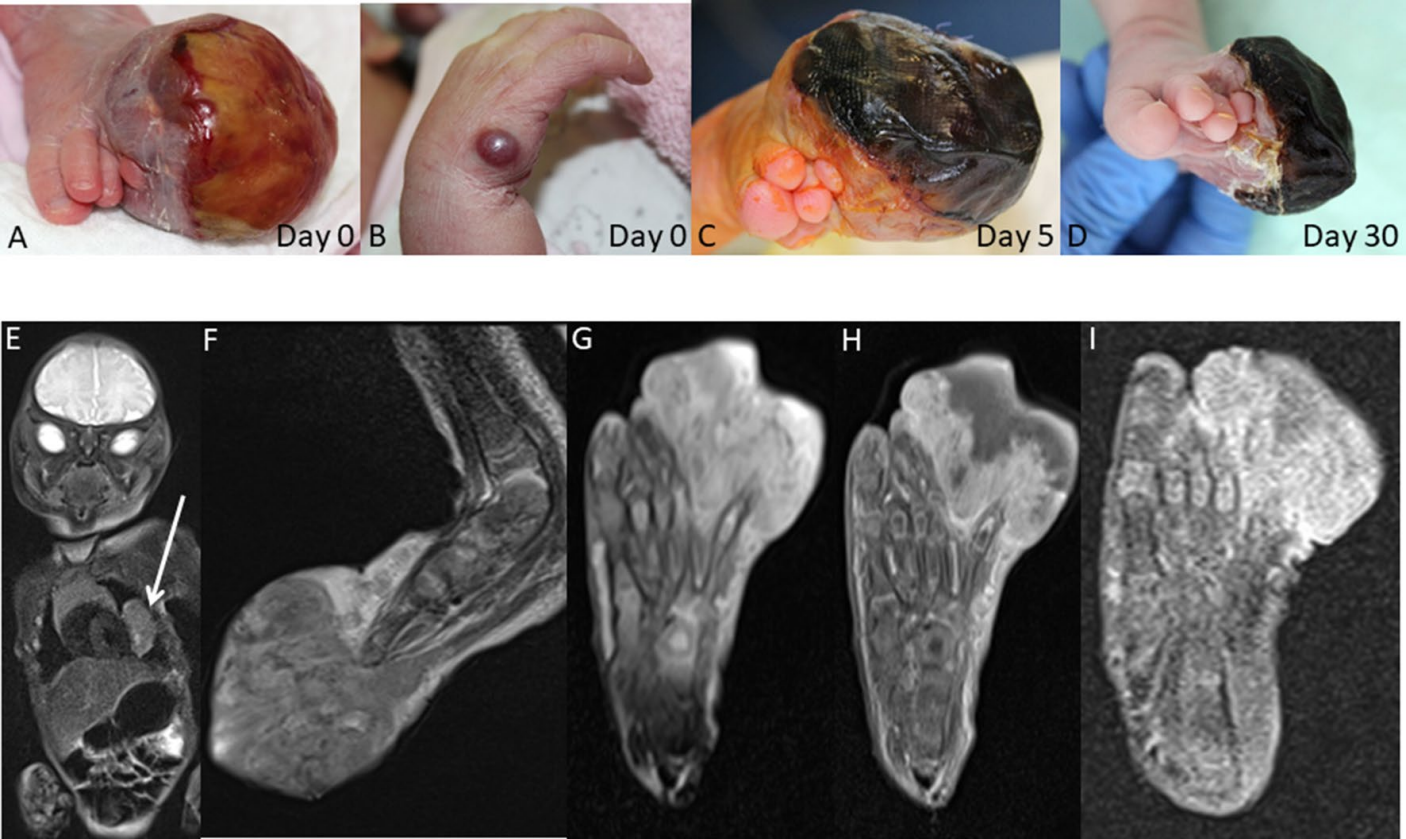
Familial IM manifesting with multifocal lesions at birth. **a** Large tumor on left forefoot immediately after birth. **b** Mass on right hand immediately after birth. **c** Necrosis of the tumor on the left forefoot on day of life 5. **d** Further shrinkage of the tumor on the left forefoot on day of life 30. **e** Left paracardial mass and **f**, **g** large tumor on left forefoot depicted by whole body MRI on day of life 4. **g**, **i** Shrinkage of the tumor on the left forefoot as illustrated by serial MRI imaging on **g** day of life 4, **h** day of life 6 and **i** day of life 26

A female infant, born at 38 + 3 weeks gestation by vaginal delivery to a 27 year-old healthy mother, presented with a large round mass on the left forefoot and a 9 mm red-colored lesion on the lateral aspect of the right hand at birth (Fig. 1a, b). Prenatal screening had not revealed any anomalies. Whole body MRI was carried out on day of life 4 and demonstrated contrast-enhancing lesions in the chest (2.6 × 2.4 × 1.4 cm, located in the left paracardial region with direct contact to the thymus, Fig. 1e), on the left foot, right hand and left-sided rectus femoris muscle. High resolution MRI of the left foot revealed a 4.3 × 4.2 × 2.4 cm mass on the left forefoot with bony involvement, complete enclosure of the 5th toe, 180° enclosure of the 4th toe and partial necrosis (Fig. 1f, g). Open incisional biopsies of the lesions on the left forefoot and right hand were carried out on day 5 of life. Histology was consistent with IM. It was decided to take a wait-and-see approach. The child developed new tumors on the left temporal aspect of the head and the left thigh within 35 days, but the mass on the left forefoot continued to shrink (Fig. 1a, d, g–i), the lesion on the right hand disappeared completely, and the intrathoracic tumor remained stable without signs of progression. The child was 1.5 months old at the time of this report and clinically well with persistent lesions on the head, thigh, left foot and in the chest. Of note, the child’s older sister had presented with a right cervical mass at the age of 5 weeks. At 2 months of age, an additional mass developed on the left thigh. Both tumors were removed by incisional biopsies and consistent with myofibromas. The sister’s subsequent clinical course was uneventful. Taken together, these two cases illustrate familial manifestation of IM with multifocal lesions, including a paracardial mass in the younger sister, within the first month of life. Written informed consent regarding this report was obtained from the parents.

### *PDGFRB* variants in infantile myofibromatosis

#### Germline variants

Variants in the platelet-derived growth factor receptor-beta (*PDGFRB*) gene were initially discovered in families with autosomal-dominantly inherited IM [3, 4]. At least 20 families have been reported so far, 19 of which carry a heterozygous *PDGFRB* variant (NM_002609.4): p.Arg561Cys in 15 (79%) of these 19 cases. The same variant was also reported in sporadic cases of IM [5–7]. Three families carry unique *PDGFRB* germline variants that are classified as likely pathogenic: p.Pro560Leu, p.Arg561Ser, and p.Lys567Glu [8–10]. All these variants cluster in the juxtamembrane domain of the receptor, encoded by exon 12. One family had a p.Pro660Thr variant of uncertain significance in the kinase domain, encoded by exon 14 [4, 11]. In one family with wild type *PDGFRB,* a heterozygous *NOTCH3* variant of uncertain significance (i.e., NM_000435.2: p.Leu1519Pro) was reported [4].

To our best knowledge, 44 individuals were reported in the literature to carry a germline *PDGFRB* variant [3–6, 8-10, 12–14]. Clinical information is available on 36 of these 44 individuals (Table 1). The phenotype of individuals within the same family may vary considerably, from asymptomatic carriers to lethal generalized IM [10, 14]. Four out of 36 (11%) *PDGFRB* variant carriers, whose clinical course was described in the literature, were IM-free (Table 1). Yet, incomplete penetrance remains difficult to quantify because benign isolated nodules may go unnoticed and regress spontaneously in childhood [10, 13]. Three out of 36 (8%) *PDGFRB* variant carriers developed solitary IM, 29 (81%) were diagnosed with multicentric or generalized disease, and 2 (6%) died. Data on age at diagnosis are incomplete, but the majority of *PDGFRB* germline variant carriers were diagnosed with IM during infancy/early childhood. There was at least one case of congenital IM (diagnosed during the first month of life) in 7 of 13 families (Table 1).

**Table 1:** PDGFRB germline variants in families with heritable IM reported in the literature

Family^a^	References	PDGFRB variant	No. of family members with IM^c^	No. of PDGFRB variant carriers per family					Age at diagnosis of IM^b^
				Total	w/solitary IM	w/multicentric or generalized IM	Asymptomatic	Dead of disease	
1	[3]	p.Arg561Cys	3	3	1	2	0	0	6–48 mo
2	[3]	p.Arg561Cys	2	2	0	2	0	0	< 12 mo
3	[3]	p.Arg561Cys	3	3	0	3	0	0	< 48 mo
4	[3]	p.Arg561Cys	3	3	0	3	0	0	1–7 mo
5	[13]	p.Arg561Cys	2	3	0	2	1	0	0–5 mo
6	[9]	p.Pro560Leu	6	5	0	4	1	0	< 1mo
7	[10]	p.Lys567Glu	5	6	1	4	1	1	0–12 mo
8	[10]	p.Arg561Cys	3	3	1	2	0	0	0–14 mo
9	[8]	p.Arg561Ser	3	2	0	2	0	0	0–12 mo
10	[12]	p.Arg561Cys	9	2	0	2	0	0	0-12mo
11	[14]	p.Arg561Cys	1	2	0	1	1	1	< 1 mo
12	[6]	p.Arg561Cys	1	1	0	1	0	0	< 25 mo
13	[5]	p.Arg561Cys	1	1	0	1	0	0	19 yo
Total			42	36	3	29	4	2	

*No of* number of, *w/* with, *IM* infantile myofibromatosis,

^a^Martignetti et al. reported 7 more families with p.Arg561Cys PDGFRB variants and 1 family with a p.Pro660Thr PDGFRB variant and IM without providing further clinical information. We included two isolated germline cases without familial history

^b^Information on age at diagnosis of IM is available on a subset of patients only

^c^This column includes cases that were not genotyped

#### Somatic variants

In the largest series of sporadic cases analyzed so far, Dachy and colleagues found somatic *PDGFRB* variants in 68% of IM and 29% of isolated solitary pediatric IM [7]. In multicentric disease, the same post-zygotic variant can be found in different nodules that develop independently in the same patient, suggesting constitutional mosaicism. In about half of the cases, *PDGFRB* contains double mono-allelic variants: a first one in exon 12, which may be germline or somatic, is associated with a second one in exon 14, such as p.Asn666Lys [3, 7, 15, 16]. RNA analysis revealed that the two variants are located on the same allele, the other allele being wild type [15]. This is reminiscent of the pathogenic variants found in another receptor tyrosine kinase gene, *TIE2*, in venous malformations [17]. Finally, a complex somatic/mosaic *PDGFRB* re-arrangement with an apparent partial tandem duplication involving the juxtamembrane domain and resulting in MAPK activation was recently detected in a newborn with multicentric IM [18].

#### Functional impact

**Fig. 2:**
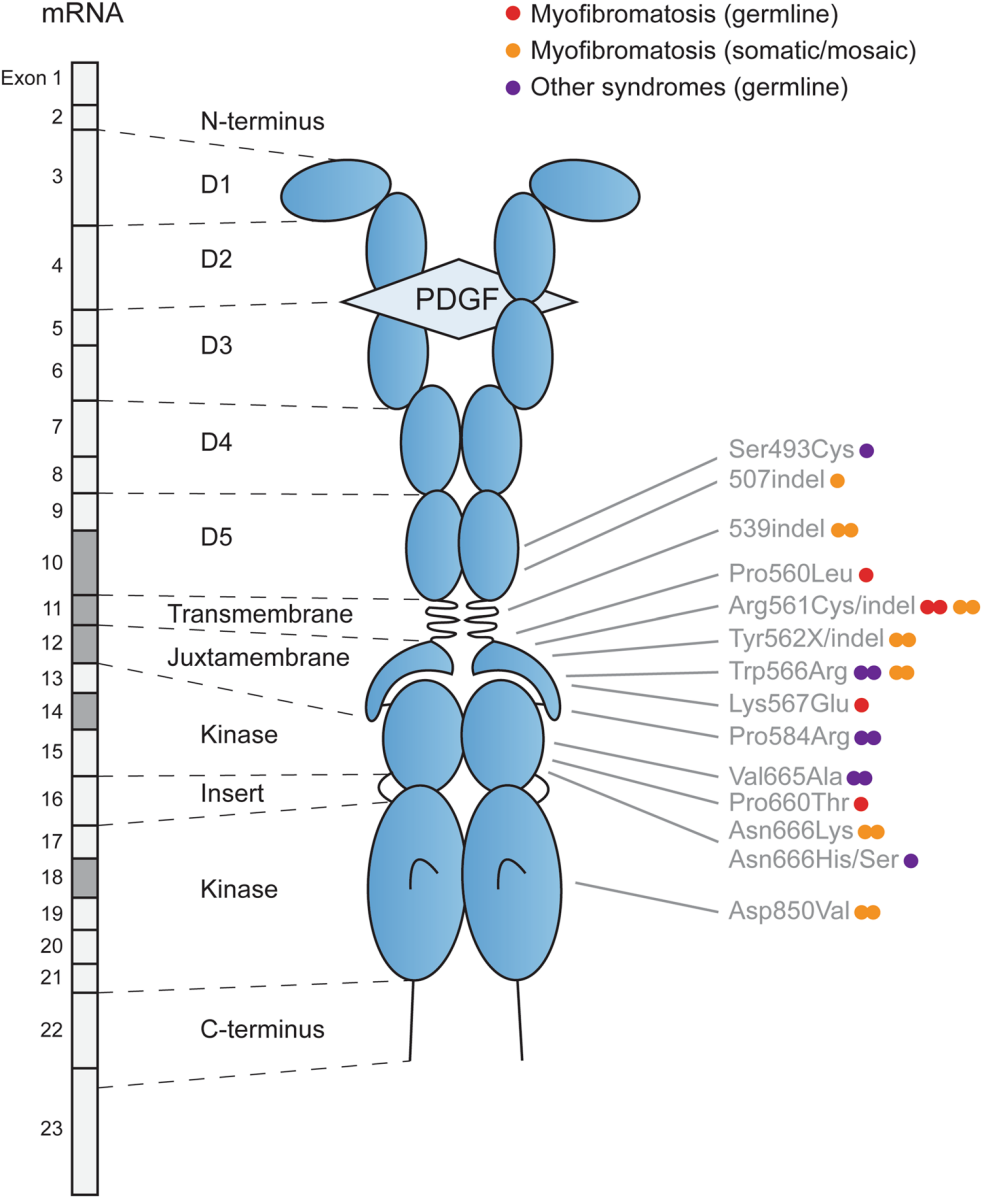
*PDGFRB* variants in infantile myofibromatosis and other diseases. The location of all germline and most significant somatic/mosaic variants of *PDGFRB* is indicated, with the corresponding exons in dark grey (NM_002609.4). Orange and red dots depict IM mutations that are somatic/mosaic and germline, respectively. Purple dots correspond to gain-of-function mutations found in other diseases (Kosaki overgrowth, Penttinen, and related syndromes). Double dots indicate recurrent mutations. “Indel” indicates the position of reported small in-frame insertions and deletions. X denotes that several amino-acid substitutions were reported at the indicated position. Loss-of-function mutations associated with Fahr disease are not shown. D1 to D5: extracellular Ig-like domains of the receptor

Appropriate signaling though PDGFRB is essential for a variety of cells such as radial glia, renal glomerular cells as well as for pericytes, which are the proposed cells of origin of IM. *PDGFRB* variants associated with IM constitutively activate PDGFRB receptor kinase activity [11]. Most variants are located within the juxtamembrane domain or in the kinase domain, the function of which is to prevent inappropriate activation of the kinase. Nevertheless, pathogenic variants are also found in the extracellular and transmembrane domains of the receptor. Interestingly, germline variants cluster in the juxtamembrane domain (Fig. 2). Their constitutive activation capabilities are weaker than those of somatic variants, and double mutant alleles frequently displayed additive activation in reporter assays, thus indicating the cooperating effects of germline and somatic variants affecting the same allele [15].

#### Imatinib sensitivity

Imatinib is a potent inhibitor of the kinase domain of PDGF receptors. This drug was approved for the treatment of multiple neoplasms associated with aberrant PDGF receptor activation, including dermatofibrosarcoma protuberans, gastrointestinal stromal tumors, a subset of acute lymphoblastic leukemia, and myeloid neoplasms with hypereosinophilia [19]. Most of the mutants identified in IM are highly sensitive to imatinib in vitro [7]. Only one variant, p.Asp850Val, confers full resistance to this compound, suggesting that most patients with PDGFRB-mutant IM may be eligible for imatinib therapy. Two case reports suggest that this drug is effective and well tolerated [5, 20]. Side effects include growth retardation, as reported in children with chronic myeloid leukemia treated with imatinib [21].

### Genetically related disorders with and without myofibroma susceptibility

The clinical phenotype associated with *PDGFRB* germline variants is variable (Fig. 2). Germline *PDGFRB* mutations can also cause other congenital diseases, including Kosaki overgrowth syndrome (OMIM 616592) and Penttinen syndrome (OMIM 601812), reported in a small number of patients. Germline gain-of-function variants associated with these conditions activate PDGFRB more potently than those found in familial IM [11].

Kosaki overgrowth syndrome is associated with tall stature, dysmorphic facial features, hyperelastic skin, and progressive neurological deterioration, mostly associated with white matter changes [22, 23]. The clinical features of Kosaki overgrowth syndrome include myofibromas. Patients with Penttinen syndrome exhibit premature aging, lipoatrophy, dermal atrophy, and thin hair [24, 25]. *PDGFRB* mutations associated with mixed phenotypes were reported [20, 26].

Somatic/mosaic activating variants within the juxtamembrane domain or the kinase activation loop were also described in 4 out of 6 patients with fusiform cerebral aneurysms [27]. In fact, recent studies suggest that aneurysms may be common in patients with germline *PDGFRB* variants (e.g. p.Trp566Arg, p.Ser493Cys) and cause severe complications, including sudden death [15, 28–30]. Aneurysms were also reported in a sporadic IM case [15].

Finally, heterozygous loss-of-function variants of *PDGFRB*, among other genes, have been associated with primary familial brain calcification (also named idiopathic basal ganglia calcification 4 or Fahr disease, OMIM 615007). This condition features a bilateral calcification of the basal ganglia, and neurological symptoms developing throughout life, including Parkinsonism, impaired cognitive function, migraine, and depression [31].

### Other genes in infantile myofibromatosis

Martignetti and colleagues identified a germline *NOTCH3* variant in all affected individuals of a single family with IM [4]. However, functional characterization of the variant is still lacking and germline *NOTCH3* variants have not been reported in other families or patients with IM. Using RNA sequencing of tumor samples, Antonescu and colleagues reported somatic serum-response factor (*SRF*) fusion genes in IM, including *SRF-RELA* [32]. *SRF* encodes a transcription factor that is controlled by mitogen signaling pathways, downstream PDGF receptors, and has been shown to be involved in murine heart and vascular smooth muscle cell development [32]. Future studies should address the functional consequences of *SRF* fusion genes and establish whether *SRF* fusions and *PDGFRB* alterations are mutually exclusive.

## Brief review of the current literature

### Infantile myofibromatosis

#### Clinical manifestation

IM commonly presents with firm, non-tender, flesh-colored nodules, which may arise in any body region. IM lesions are most commonly located in the skin, subcutaneous tissue or muscle of the head, neck, and trunk. Superficially located nodules are often noted by parents and caregivers and are non-ulcerative. Deep-seated foci are either detected by imaging following recognition of superficial lesions or through their symptomatology. Up to 74% of all IM cases are detected shortly after birth; 89% of IM cases are diagnosed within the first two years of life [33]. Rare first presentations of IM in older children and adults have been reported in the literature [5]. IM presents with one of three clinical patterns: solitary (50 to 74% of all cases), multicentric, and generalized (with visceral involvement) manifestations. Skeletal lesions are common in children with multicentric IM (approximately 75% of cases [2, 34, 35]). Multicentric IM may present with or without visceral involvement. Visceral IM lesions have been observed in 11 to 19% of cases [2, 33, 36]. Visceral lesions typically involve the cardiopulmonary and/or gastrointestinal system. Intracranial (both intra- and extra-axial) involvement has been seen occasionally [37]. Familial IM cases follow an autosomal-dominant mode of inheritance, and often present with early-onset multicentric lesions [2, 3].

#### Diagnosis

Histopathologically, tumors are nodular and composed of myoid spindle-shaped cells with pink cytoplasm and elongated nuclei without atypia, arranged in a fascicular pattern with varying cellularity and myointima-like fibromyxoid aggregates (so-called vascular balls). Mitotic activity is usually low or minimal, but mitotically active lesions may be encountered, particularly in so-called atypical IM [38]. IMs frequently exhibit a characteristic hemangiopericytoma-like vascular pattern and may show extensive hyalinization or other regressive features, including hemorrhage, cystic degeneration, calcification, and even necrosis, which may give a false impression of malignancy. Tumor cells are often positive for vimentin and smooth muscle actin, but they are usually negative for desmin. S100, epithelial membrane antigen, keratin, and vascular markers are absent in tumor cells [39]. All patients diagnosed with IM are recommended to be screened radiologically for multicentric disease and visceral involvement.

#### Outcome

IM may regress spontaneously, typically within 18 to 24 months after diagnosis. Lesions may leave atrophic scars. Recurrences are possible. Visceral involvement is generally considered a poor prognostic feature. Mortality rates up to 76–93%, often due to cardiopulmonary or gastrointestinal complications, were observed in published cohorts of 28 to 31 children with multicentric disease with multiple visceral IM lesions [33, 36, 40]. Of note, aneurysms and fibromuscular dysplasia were reported in patients after a prior diagnosis of sporadic IM [15, 41–43]. A few children with sporadic IM were also reported to develop malignant tumors in addition to IM: fibrosarcomatous transformation of a congenital solitary IM was observed in a 14-months-old child [44]. Features of high-grade malignancy were also seen in a recurrent lesion at the base of the tongue approximately 10 years after first diagnosis of a IM in the same area [45]. Metastatic rhabdomyosarcoma was diagnosed in a 2-year-old girl after spontaneous resolution of IM with visceral involvement [2]. A “neuroblastoma of the brainstem” was diagnosed in a 3-months-old child with a prior diagnosis of multicentric IM with orbital, facial, and intraoral lesions [46]. It is not clear if the malignant diagnoses in these anecdotal cases were confirmed by reference histology review or substantiated by further molecular analysis.

#### Treatment

Many patients diagnosed with IM have little or no symptoms. Disease progression is typically slow and local. A wait-and-see strategy is appropriate for many patients. If resection without sequelae appears feasible, solitary lesions may be removed surgically [2, 33, 35]. Patients with visceral involvement should be followed closely given the high risk of associated morbidity and mortality [33, 36, 40]. Systemic therapy is only recommended in case of life-threatening progressive disease, typically due to compression of vital structures or organ dysfunction in the setting of progressive visceral disease [35]. Vincristine/dactinomycin and vinblastine/methotrexate regimens have been used with good results in patients with IM [35, 47]. Yet, similar to what has been observed in patients with aggressive fibromatosis [48–50], response to treatment tends to be slow. Alternatively, a few children with *PDGFRB*-mutated IM have been treated successfully with imatinib and sunitinib [8, 15, 20, 28]. Prior to initiating systemic therapy, the acute and long-term side effects should always be considered carefully and weighed against the expected benefits.

## Consensus recommendations

IM is a rare disease. The published literature is based on small cohorts [2, 34, 35]. The following recommendations summarize a review of the current literature and expert discussions within the SIOPE Host Genome Working Group.

### Diagnosis

Prior to any biopsy or surgical intervention, it is important to rule out fibrodysplasia ossificans progressiva (FOP, OMIM 135100), which is typically associated with an easily recognizable congenital valgus malformation of the big toes in affected infants [51]. FOP is a very rare condition caused by germline variants in the gene encoding activin A receptor type 1 (*ACVR1*) and is associated with progressive heterotopic extraskeletal ossification, which may present as tumor-like masses typically in the shoulder and back areas, on the scalp or head. Any type of soft tissue injury, including surgical removal or biopsy of soft-tissue lesions, may stimulate the development of new ossification foci. For all other children with a suspected diagnosis of IM, biopsy and verification of histopathology diagnosis by reference histology review is strongly recommended. All children diagnosed with IM (including solitary IM) should be screened for multicentric disease using whole body MRI and/or PET [52]. Cardiac lesions should be ruled out by cardiac ultrasound. Abdominal ultrasound, low-dose chest CT, skeletal surveys, and MRI of the brain may also be used to detect associated lesions [34]. For all patients with suspected IM, a careful family history covering three generations should be taken and specifically include history of small soft tissue nodules.

### Genetic counseling and germline genetic testing

Genetic counseling and germline analyses of *PDGFRB* should be discussed with the parents of/offered to all children diagnosed with IM and at least one of the following criteria: (i) multicentric manifestation, (ii) ≥ 1 first or second degree relatives with IM or a history of soft tissue nodules during childhood, (iii) a known causal or likely causal germline *PDGFRB* variant in the family, or (iv) suspected germline (mosaic) *PDGFRB* variants. Since the vast majority of *PDGFRB* germline variants are located in exon 12, which encodes the juxtamembrane domain, Sanger or NGS sequencing of that exon would be appropriate in most cases and reduce the number of detected variants of uncertain significance. However, it seems to be more realistic to get a virtually trimmed diagnostic *PDGFRB* (exon 12) sequencing result given the general switch to automated next-generation sequencing approaches technically covering gene panels, exomes, or even genomes. Predictive testing of relatives at risk can be offered to allow for identification of affected individuals and implement expert-based recommendations for surveillance during childhood.

### Sequencing of *PDGFRB* in tumor tissue

IM has been associated with pathogenic variants in *PDGFRB*, which may confer sensitivity to tyrosine kinase inhibitors such as imatinib. We recommend testing of IM tumor tissue for the presence of *PDGFRB* mutations by next-generation sequencing (NGS). Most variants map to exons 12 and 14, suggesting that sequencing of these two hotspots may be sufficient in many cases. Nevertheless, up to one third of the reported pathogenic variants are located in other exons. Whenever possible, *PDGFRB* deep sequencing should be performed to increase sensitivity [7]. If disease is multicentric and no mutation is detected in blood-derived DNA, we recommend testing at least three lesions (if possible) for the presence of *PDGFRB* mutations to screen for mosaicism.

### Functional testing of *PDGFRB* variants

*PDGFRB* variants detected in IM tissue have been linked to sensitivity to tyrosine kinase inhibitors, including imatinib. A list of imatinib-sensitive *PDGFRB* variants, previously detected in IM tissue, has been provided by Dachy et al [7]. Imatinib-sensitivity testing of other, new *PDGFRB* variants should be considered whenever possible.

### Surveillance recommendations

IM presents mostly early in life and is generally associated with excellent outcomes. However, multicentric disease with visceral involvement, which is typically diagnosed in young infants, has a rather high mortality. Our review of the literature, indicates that IM risk in *PDGFRB* variant carriers is high, and many patients with familial IM are diagnosed with multicentric/generalized disease at birth or during infancy (Table 1). Additional features associated with *PDGFRB* germline variants include cerebral aneurysms. Knowledge of a family history of IM or familial *PDGFRB* variants may cause substantial anxiety.

Taking these considerations into account, patients with a first-degree relative with IM (solitary, multicentric or generalized) or a proven *PDGFRB* germline variant should undergo a baseline physical examination, abdominal and cardiac ultrasound after birth or following the detection of a causal familial *PDGFRB* germline variant. Follow-up clinical evaluation, including a complete physical examination, evaluation of growth/weight gain, blood pressure measurements and abdominal ultrasound, is recommended every 3 months until the age of 24 months, then once every 1 to 2 years until the age of 12 years. Any suspicious physical findings such as soft-tissue nodules, heart murmurs and failure to thrive should prompt further diagnostic evaluation, including whole body MRI and cardiac ultrasound (Table 2).

**Table 2:** Surveillance recommendations for individuals with a proven *PDGFRB* variant or first-degree relative with IM

Age	Surveillance recommendations	Timing
At birth or after first detection of *PDGFRB* germline variant	Genetic counseling Physical examination Abdominal ultrasound Cardiac ultrasound	Once
Age 0–24 months	Physical examination Evaluation of growth/weight gain Abdominal ultrasound	Every 3 months
Age 2–12 years	Physical examination Evaluation of growth/weight gain Abdominal ultrasound	Yearly
Age 15–18 years	MRI brain	Once

Any suspicious physical findings such as soft-tissue nodules, heart murmurs and failure to thrive should prompt further diagnostic evaluation, including whole body MRI and cardiac ultrasound. Genetic counseling should be offered to all patients/parents of patients with IM with a proven *PDGFRB* variant or a family history of IM

As *PDGFRB* germline variants associated with IM were recently also linked to the development of cerebral aneurysms, a single MRI examination of the brain at the age of 15–18 years to screen for cerebral aneurysms can be considered.

Many patients with familial IM are diagnosed with multicentric or generalized disease during early infancy. Prenatal screening by ultrasound and, if there are any suspicious findings, by prenatal MRI may be considered during the third trimester, if one parent carries a *PDGFRB* germline variant (typically p.Arg561Cys).

## Research needs

We call for systematic registration of patients with IM to obtain a better understanding of the molecular underpinnings of IM and the course of disease and genotype–phenotype correlations.

Important clinical questions include:

1. Prevalence of *PDGFRB* germline and somatic variants and *PDGFRB* mosaicism in children diagnosed with IM.
2. Possible differences in IM penetrance in patients with different *PDGFRB* germline variants.
3. Rate of visceral disease, life-threatening complications and death in patients with *PDGFRB* germline variants, *PDGFRB* somatic/mosaic variants and those without *PDGFRB* variants.
4. Associated medical issues in *PDGFRB* germline variant carriers, including issues developing later in life, which may include aneurysms/fibromuscular dysplasia, malignant tumors, and neurological abnormalities.

Important biological questions include:

1. Correlation of specific *PDGFRB* variants with imatinib sensitivity.
2. Mechanism of development of somatic *PDGFRB* variants on the allele with the germline *PDGFRB* variant.
3. Multicentric/generalized IM is generally considered a multifocal disorder. Metastases have anecdotally been reported in the past [53]. It is conceivable that certain lesions represent metastases rather than multiple independent foci, but further clonality analyses/molecular testing are needed to understand the origins of multicentric disease.
4. Pathogenicity of other, non-*PDGFRB* aberrations in IM.
5. Potential overlap between myofibromas and myopericytomas, benign perivascular tumors of pericytes, which bear close resemblance to myofibromas [32].
6. Definition of the IM cell of origin.

## Conclusions

IM is an extremely rare disease, which is typically diagnosed in young children and comprises a wide clinical spectrum ranging from solitary soft tissue nodules causing few symptoms to multiple disseminated tumors resulting in life-threatening complications. Based on interdisciplinary discussions within the SIOPE Host Genome Working Group, we provide recommendations for somatic and germline genetic testing, genetic counselling, and surveillance. Close international and interdisciplinary collaboration is needed to improve our understanding of the clinical course and genetic underpinnings of IM and refine clinical practice recommendations.
